# Computed Tomography Radiomics Nomogram to Predict the Intraoperative Hypertensive Crisis of Abdominal Pheochromocytoma and Paraganglioma

**DOI:** 10.2174/0115734056320071241120090524

**Published:** 2025-01-17

**Authors:** Qianru Zhang, Xu Fang, Liangping Ni, Li Wang, Jianping Lu, Chengwei Shao, Yun Bian

**Affiliations:** 1 Department of Radiology, Changhai Hospital, Naval Medical University, Shanghai, China; 2 Department of Radiology, The Second Affiliated Hospital of Anhui Medical University, Hefei, China

**Keywords:** Pheochromocytoma, Paraganglioma, Radiomics, Hypertension, X-ray computed tomography

## Abstract

**Background:**

Patients with abdominal Pheochromocytoma and Paraganglioma (PPGL) are prone to a hypertensive crisis during surgery, which may endanger their lives. This study aimed to develop and validate a Computed Tomography (CT) radiomics nomogram for the prediction of intraoperative hypertensive crisis in patients with PPGL.

**Methods:**

In this retrospective study, 212 patients with abdominal PPGL underwent abdominal-enhanced CT and surgical resection. Radiomic features were extracted from arterial and venous phases. Multivariable logistic regression models were developed using an internal validation and an external test set. The performance of the nomograms was determined by their discrimination, calibration, and clinical usefulness.

**Results:**

A total of 212 patients with PPGL were included, involving 44 with hypertensive crises. The patients were divided into training (n = 117), validation (n = 51), and test (n = 44) sets. Eighteen radiomics-relevant radiomic features were selected. A history of coronary heart disease and the CT radiomics score were included in the prediction model, which achieved an area under the curve of 0.91 [95% Confidence Interval (CI) 0.85-0.97] in the training set, 0.93 (95% CI 0.84-0.99) in the validation set, and 0.85 (95% CI 0.72-0.97) in the test set. The decision curve analysis demonstrated the radiomics nomogram to be clinically useful.

**Conclusion:**

Our study has developed and validated a CT radiomics nomogram that has demonstrated remarkable potential in predicting intraoperative hypertensive crisis in patients with abdominal pheochromocytoma and paraganglioma. This non-invasive, straightforward approach has exhibited high accuracy, ease of use, and predictive power.

## 1 INTRODUCTION

Pheochromocytomas and Paragangliomas (PPGLs) constitute a group of neuroendocrine tumors renowned for their production of one or more catecholamines [1]. It is estimated that approximately 80-85% of PPGLs originate from the adrenal medulla, while 75% of extra-adrenal paragangliomas are found within the abdominal cavity [2]. Surgical resection stands as the cornerstone of treatment for PPGLs [1]. However, this surgical intervention is acknowledged as high-risk due to the potential for anesthetic induction or intraoperative tumor manipulation to elicit an uncontrollable surge of catechola-mines, thereby precipitating life-threatening cardiovascular complications, including severe hypertensive crises and arrhythmias [2-4]. Historically, hypertensive crises have been implicated in perioperative mortality rates reaching up to 48% among adult patients [5].

Contrast-enhanced Computed Tomography (CT) remains the gold standard imaging modality for evaluating Paragangliomas and Pheochromocytomas (PPGLs), offering unparalleled insight [1]. A distinguishing CT feature of PPGLs is the conspicuous presence of an intensified solid mass, which underscores their identity [6]. While a symbiotic fusion of distinct imaging attributes and clinical manifestations bolsters preoperative diagnostic certainty, conventional CT alone falls short in anticipating the likelihood of an intraoperative hypertensive crisis. Radiomics, an emerging field, has demonstrated immense potential in preoperatively characterizing and differentially diagnosing PPGLs, further refining diagnostic acuity [7-9]. Concurrently, there exists a corpus of research exploring the combined prowess of clinical manifestations, such as coronary heart disease, and CT imaging findings to foresee hypertensive crises in PPGL patients [10-14]. Nonetheless, the frontier of predictive accuracy has yet to be breached by leveraging radiomics models solely for this purpose. Motivated by these seminal observations, we hypothesize that CT-derived radiomic signatures hold immense promise as biomarkers for predicting the occurrence of intraoperative hypertensive crises in abdominal PPGL patients. Such a precise preoperative prognosis would empower surgeons and anesthesiologists with the foresight to meticulously prepare preoperative measures and tailor perioperative management strategies, thereby enhancing surgical safety margins and optimizing overall outcomes.

In this study, our objective was to develop and validate a preoperative CT radiomics nomogram combined with clinical characteristics to predict the intraoperative hypertensive crisis associated with abdominal PPGL. This nomogram would incorporate radiomic signatures and clinical factors. The structure of this paper is organized as follows: section 1 outlines the materials and methods employed in this study, section 2 presents the results of our analysis, and section 3 discusses the implications and limitations of our findings.

## 2 MATERIALS AND METHODS

### 2.1 Patients

All consecutive patients admitted to Changhai Hospital between January 2011 and December 2021 and The Second Affiliated Hospital of Anhui Medical University between January 2018 and June 2022 were retrospectively reviewed. The biomedical research ethics committee of Changhai Hospital and The Second Affiliated Hospital of Anhui Medical University review boards approved this study and waived the requirement for informed consent.

### 2.2 Inclusion and Exclusion Criteria

All patients with PPGL who were pathologically confirmed were included. There are a total of 288 patients at Changhai Hospital and 76 patients at The Second Affiliated Hospital of Anhui Medical University. Patients who (a) were not evaluated via contrast-enhanced CT within the month prior to surgery, (b) had poor imaging quality, (c) had incomplete blood pressure data during surgery, and (d) had undergone surgical resection and postoperative recurrence, were excluded. As a result, 212 patients were enrolled in this study, including 168 patients treated at the Changhai Hospital and 44 treated at The Second Affiliated Hospital of Anhui Medical University. Subsequently, patients from Changhai Hospital constituted both the training and validation sets [15], whereas those from The Second Affiliated Hospital of Anhui Medical University were reserved for the external test set. The patient inclusion and exclusion criteria for this study are shown in Fig. (**1**).

### 2.3 Sample Size Estimation

Following the guidelines for sample size calculation in clinical research with binary outcomes [16], we selected an α value of 0.05, a power (1-β) of 90%, an Area Under the Curve (AUC) of 0.75, and an AUC1 of 0.90, resulting in a minimum sample size of 34 participants in each group. The sample size of this study met these requirements. Sample size estimation was done using PASS 15 Power Analysis and Sample Size Software (NCSS, LLC. Kaysville, Utah, USA; ncss.com/software/pass).

### 2.4 Clinical Characteristics

Clinical data were obtained from medical records, including the following: sex, age, Body Mass Index (BMI), “triad” symptoms (headache, palpitations, and diaphoresis), history of hypertension, diabetes, coronary heart disease, taking hypertensive drugs before surgery, operation method (laparotomy or laparoscopy), and operative time [13, 14, 17]. In this study, patients were divided into two groups: a hypertensive crisis group and a non-hypertensive crisis group. Hypertensive crisis was defined as a systolic blood pressure of ≥180 mmHg or a diastolic blood pressure of ≥120 mmHg during the operation [18].

### 2.5 CT Scan

During the study period, abdominal CT was performed using two scanners: 320-slice multidetector row CT (Aquilion ONE; Canon Medical Systems, Tokyo, Japan) and 256-slice multidetector row CT (Brilliance iCT, Philips Healthcare, Cleveland, Ohio, USA). The scanning parameters during breath holding were as follows: 120 kVp, 270 mA or auto mA mode, a 256-256 matrix, 320-380 mm FOV, and a slice thickness interval of 5 mm. Intravenous administration of iodine contrast medium was performed using an automatic power injector. The contrast agent (80-100 mL of 370 mg I/mL iopromide; Ultravist 370, Bayer Schering Pharma, Berlin, Germany) and 300 mgI/mL iohexol (Omnipaque, GE Healthcare, Milwaukee, USA) were injected at a rate depending on the patient’s body weight, followed by 20 mL of normal saline for flushing. Images were obtained during the arterial and venous phases at 30 and 70 s, respectively, after the contrast medium injection.

### 2.6 CT Imaging Analysis

We used the original cross-sectional CT images for radiological analysis. All images were analyzed by two abdominal radiologists (YB and XF, with 20 and 10 years of experience, respectively). Both radiologists were blinded to all clinical and pathological details, and the results were determined by consensus.

**Fig. (2):**
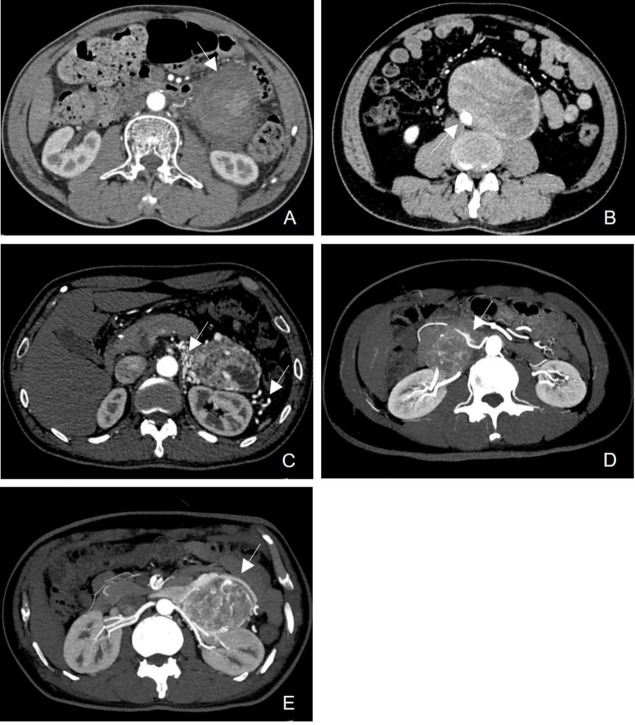
**A.** The presence of capsular invasion in a 75-year-old woman with paraganglioma. The tumor capsule was discontinuous with ill-defined margins (arrow). **B.** The presence of vascular invasion in a 30-year-old man with paraganglioma. The tumor was in contact with the abdominal aorta (arrow). **C.** The presence of collateral vessel in a 45-year-old woman with pheochromocytoma of the left adrenal gland. There were multiple asymmetrically increased and irregular arteries surrounding the tumor (arrow). **D.** The presence of a feeder artery in a 56-year-old man with paraganglioma. A conspicuous artery was observed after directly tracing the course from the tumor margin to the tumor interior for its vascular supply, which originated from the abdominal aorta (arrow). **E.** The presence of draining veins in a 45-year-old woman with pheochromocytoma of the left adrenal gland. A draining vein was observed after directly tracing the course from the tumor margin to the tumor interior for its vascular drainage, which drained into the left renal vein.

All tumors were evaluated for the following characteristics: (a) tumor location, adrenal or non-adrenal; (b) tumor number, 1 or ≥2; (c) tumor size, the maximum diameter of the tumor in a cross-section; (d) tumor shape, rounded or lobulated; (e) CT attenuation values of the tumor, which were obtained by measuring the density of the tumor in the arterial and venous phases using Regions of Interest (ROIs). The ROIs were placed above the solid component of the tumor. The attenuation value of the ROI was measured three times and then averaged; (f) cystic degeneration ratio, <50% or ≥50%; (g) calcification; (h) the presence of capsular invasion (Fig. **2A**), which was defined as discontinuous capsules with ill-defined margins [19]; (i) the presence of vascular invasion, which was defined as tumor contact with peritumoral vessels >180° (Fig. **2B**); (j) collateral vessels (Fig. **2C**), which were defined as asymmetrically increased and irregular vessels surrounding the tumor. Collateral arteries and veins were identified on the arterial and venous phases images, respectively [20]; (k) feeder artery (Fig. **2D**), which was defined as a conspicuous artery capable of directly tracing the course from the tumor margin to the tumor interior for its vascular supply on the arterial phase images [21]; and (l) draining vein (Fig. **2E**), which was defined as a conspicuous vein capable of directly tracing the course from the inside of tumor to the outside of tumor for its vascular drainage on the venous phase images [21]. When more than one tumor coexisted, the largest tumor was defined as a representative tumor.

### 2.7 Radiomics Workflow

**Fig. (3):**
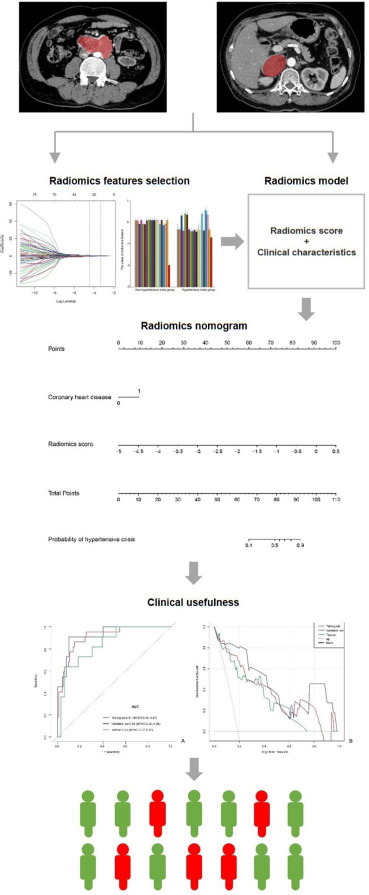
The workflow of the model.

The workflow included the following steps (Fig. **3**): (a) tumor delineation, (b) data preprocessing, (c) feature extraction, and (d) feature reduction and selection [22].

### 2.8 Tumor Delineation

The tumor Volume of Interest (VOI) was manually delineated slice-by-slice on arterial phase and venous phase CT images by a radiologist. ROI segmentation was blindly performed by two radiologists (QZ and XF) to assess interobserver reliability. XF repeated the extract feature twice over a seven-day period to evaluate intra-observer reliability. The reader completed the segmentation of the remaining images for 14 days. Interobserver and intra-observer reliability assessments were evaluated by calculating the Intra-class Correlation Coefficient (ICC). ICC values >0.75 were selected for subsequent investigations. The drawing tool available in the Editor module of 3D Slicer version 4.8.1 (open-source software; https://www.slicer.org/) was used to delineate tumors in the CT slices.

### 2.9 Data Preprocessing

There were four steps involved in the CT image preprocessing for the deep learning algorithm, including format conversion, resampling, normalization, and data augmentation. (1) Format conversion: To satisfy the training process using the 3D-UNet deep learning method, we converted the DICOM images of each phase to the Neuroimaging Informatics Technology Initiative (NIFTI) format [23] files using Python 3.7 with the dicom2nifti package [24]. Next, the 3D-UNet deep learning method was employed to make use of CT images by converting these 3D images into a 3D array. (2) Resampling: Different CT scanner products and acquisition protocols may result in inconsistent voxel spacing. Voxel spacing is essential for a CNN-based model to learn spatial semantics. Therefore, we calculated the median voxel spacing of the entire dataset and resampled all CT images using nearest-neighbor interpolation. (3) Normalization: Since the intensity scale of CT scans is absolute, we normalized all CT images by calculating the statistics of the CT image dataset. Specifically, all intensity values were obtained from CT images. The entire dataset was normalized by clipping to the 0.5, 99.5 percentiles of these intensity values, followed by z-score normalization based on the mean and standard deviation of all collected intensity values.

### 2.10 Feature Extraction

Feature extraction was performed using the open-source Python package Py 1.2.0 (http://www..io/py.html) [25]. Original and filtered were two classes of feature extraction methods used. The filter class involved 7 categories: logarithm, exponential, gradient, square, square root, lbp-2D, and wavelet [22]. In all, 1408 radiomic features were extracted from the primary tumors.

### 2.11 Feature Reduction and Selection

Variance analysis, Spearman correlation analysis, and Least Absolute Shrinkage and Selection Operator (LASSO) logistic regression algorithm analysis were the three steps employed for feature selection. Then, a retrospective power analysis was performed. To avoid the possibility of multiple test bias, we adjusted the baseline significance level (α = 0.05) using sequential Bonferroni correction [26, 27]. A linear combination of the selected features was used, combined with coefficient weighting, and finally, the radiomic score was calculated for each patient.

### 2.12 Statistical Analysis

Distribution and variance homogeneity tests were performed for all continuous variables. Normally distributed data have been expressed as means and standard deviations, whereas non-normally distributed data have been expressed as medians and ranges. The patients were divided into a hypertensive crisis group and a non-hypertensive crisis group. First, the inter-group differences were examined for all variables. Student t-test was used for normal distribution, Kruskal-Wallis H test for skewness distribution, and Chi-square test for categorical variables in order to determine whether there were statistical differences between the non-hypertensive crisis group and the hypertensive crisis group. Secondly, we conducted a univariate regression analysis on the training set to explore the correlation among clinical characteristics, imaging characteristics, radiomic scores, and hypertensive crisis. Furthermore, relevant features identified in the univariate analysis (*p* <0.05) were incorporated into a multivariate regression model to establish a predictive model for hypertensive crisis and the model was visualized as a nomogram. A stepwise regression method based on Akaike information criteria was used to determine the best-fitting model [28-30]. The discriminant ability of each model was quantified by estimating the corresponding Receiver Operating Characteristic (ROC) curve and Area Under the Curve (AUC). Finally, to estimate the clinical usefulness of the nomogram, a decision curve analysis was performed by calculating the net benefits for a range of threshold probabilities [22].

Statistical significance was set at a two-tailed p-value <0.05. All analyses were performed using the R software (version 3.3.3; R Foundation for Statistical Computing, Vienna, Austria).

## 3 RESULTS

### 3.1 Clinical and CT Characteristics

We initially enrolled 288 patients with PPGL. After exclusion, the final sample included 168 patients (median age, 50 years; 87 women) admitted to Changhai Hospital. In addition, 76 patients with PPGL were initially enrolled from The Second Affiliated Hospital of Anhui Medical University, and the final sample included 44 patients (median age, 53 years; 24 women) after exclusion. The training set comprised 117 patients, including 95 with non-hypertensive and 22 with hypertensive crisis. The validation set comprised 51 patients, including 40 with non-hypertensive and 11 with hypertensive crisis. The test set comprised 44 patients, including 33 with non-hypertensive and 11 with hypertensive crisis. In the training set, no significant differences were observed in all clinical and CT characteristics between the non-hypertensive and hypertensive crisis groups (*p* >0.05). Differences were observed in the BMI in the validation set and the presence of capsular invasion in the test set (*p* <0.05). No other significant between-group differences were observed in the internal and external validation sets (*p* >0.05). The patient characteristics are shown in Table **1**.

### 3.2 Radiomics Analysis

**Fig. (4):**
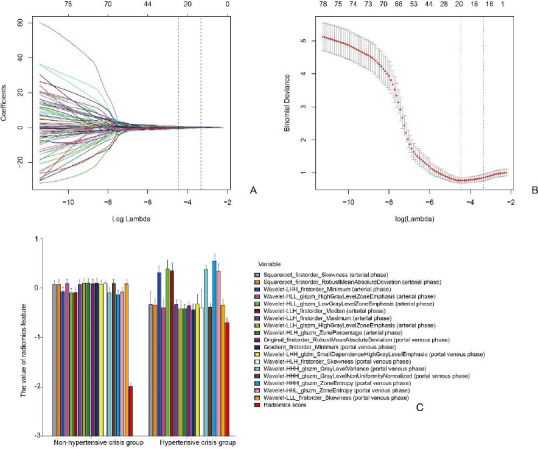
Radiomic feature selection using the parametric, Least Absolute Shrinkage and Selection Operator (LASSO) method. **A.** The tuning parameter (λ) for the LASSO model was selected through 10-fold cross-validation, based on the minimum criteria. The binomial deviance resulting from the cross-validation procedure of LASSO regression was plotted against log(λ). The y-axis represents binomial deviance, while the lower x-axis indicates log(λ) values. Numbers along the upper x-axis denote average predictor counts. Red dots represent average deviance values for each model at a given λ value, with vertical bars indicating the deviation range. Optimal λ values, where the model best fits the data, are defined by vertical black lines. An optimal λ value of 0.035 with log(λ) = -3.34 was chosen. **B.** The LASSO coefficient profiles of 78 features are presented. The vertical dotted line represents the value obtained from 10-fold cross-validation in Fig. A. In the plot, the eighteen selected features with non-zero coefficients are highlighted. **C.** An error-bar chart illustrating the relationship between these eighteen radiomics features and the radiomics score is provided.

The CT images yielded a total of 1408 extracted features. The inter- and intra-observer ICC values ranged from 0.75 to 0.82, demonstrating good agreement. Consequently, a total of 1273 radiomics features that exhibited no significant differences between the groups and showed no significant associations with non-hypertensive or hypertensive crisis were excluded from further analysis. Subsequently, the remaining set of 135 features underwent evaluation using the LASSO logistic regression model, resulting in the identification of a final selection of 18 relevant features (Fig. **4**).

### 3.3 Radiomics Model

**Fig. (5):**
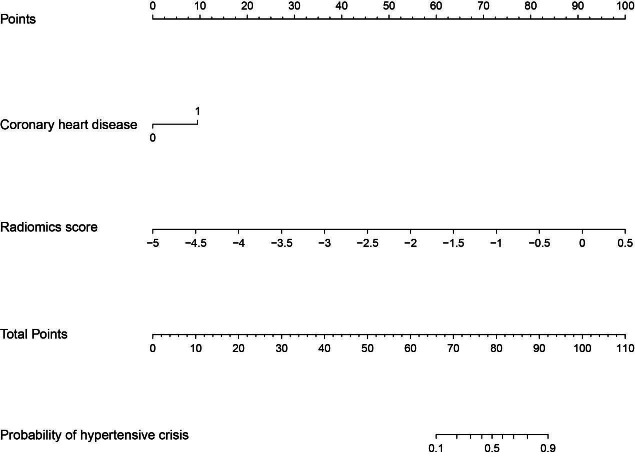
A radiomics nomogram of the training set.

**Fig. (6):**
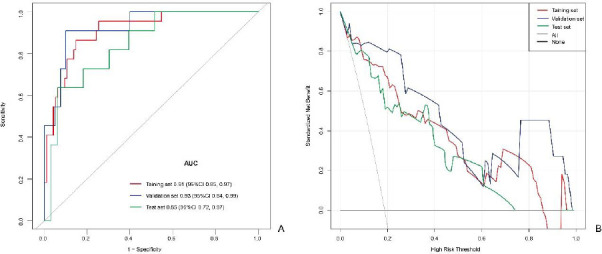
The radiomics nomogram's Receiver Operating Characteristic (ROC) curves and Decision Curve Analysis (DCA). **A.** ROC curves for the training, validation, and test sets. **B.** DCA curves for the training, validation, and test sets.

Univariate analysis indicated coronary heart disease (OR: 5.06, 95%CI: 1.16, 22.10; *p*=0.031) and radiomics score (OR: 22.20, 95%CI: 5.68, 86.75; *p*<0.0001) to be significantly associated with hypertensive crisis (Table **2**). Multivariable logistic regression analysis included the following parameters: history of coronary heart disease and CT radiomics score (Table **3** and Fig. **5**). The prediction model yielded AUCs of 0.91 [95% Confidence Interval (CI), 0.85-0.97], 0.93 (95% CI, 0.84-0.99), and 0.85 (95% CI, 0.72-0.97) in the training, validation, and test sets, respectively. The sensitivity, specificity, accuracy, positive predictive value, and negative predictive value of the model for the training set were 86.36%, 85.26%, 85.47%, 57.58%, and 96.43%, respectively, whereas those for the internal and test sets were 90.91% and 63.64%, 90.00% and 93.94%, 90.20% and 86.36%, 71.43% and 77.78%, and 97.30% and 88.57%, respectively Table **4** and Fig. **6A**). The two cases are shown in Fig. (**7**).

### 3.4 Clinical Utility

The decision curves of the radiomics model (Fig. **6B**) demonstrated that utilizing the radiomics model for predicting the non-hypertensive and hypertensive crisis, with threshold probabilities ranging from 0.05 to 0.85, yielded greater benefits compared to assuming that all patients had either a non-hypertensive or hypertensive crisis; specifically, thresholds above 0.05 for the training set and between 0.25-0.75 for the validation and test sets were obtained.

## 4 DISCUSSION

Accurate preoperative forecasting of hypertensive crisis in patients with PPGL is crucial for enabling surgeons and anesthesiologists to undertake adequate preoperative preparations and execute suitable perioperative management strategies, and ultimately, surgical outcomes. This study marks the inaugural multi-center retrospective investigation aimed at developing a preoperative CT radiomics nomogram to predict intraoperative hypertensive crisis in patients with abdominal PPGL. By integrating radiomics signature and coronary heart disease factors into the nomogram, we achieved commendable discrimination in the training (AUC 0.91), validation (AUC 0.93), and test sets (AUC 0.85), thereby demonstrating the practicability of our prediction model in furnishing vital information for clinical decision-making.

Previous research has identified risk factors for perioperative hemodynamic instability in pheochromocytoma, including urinary norepinephrine and tumor size [10-12]. Large tumors necessitate more extensive manipulation during surgical dissection and resection, resulting in high catecholamine release and hypertensive crisis [11]. These tumors secrete greater amounts of catecholamines, potentially leading to more pronounced blood pressure fluctuations during the perioperative period [31]. However, urinary norepinephrine levels and tumor size were not predictive in our study. We excluded urinary norepinephrine due to its preoperative absence in some PPGL patients, particularly those without hypertension, symptoms, or suspicion of PPGL. Regarding tumor size, we hypothesized that experienced surgeons at our institution would recognize larger tumor on preoperative CT. Consequently, patients received adequate preoperative drug regulation, and surgeons endeavored to minimize tumor manipulation during surgery, thereby mitigating catecholamine release and hypertensive crises.

Several studies have previously developed models to predict hypertensive crisis in PPGL [13, 14]. Bai *et al*. [13] developed and validated a clinical model by including 283 and 119 patients, respectively, as training and validation sets. Their model included BMI, coronary heart disease, tumor size, and preoperative use of crystal/colloid fluid to predict intraoperative hemodynamic instability in patients undergoing pheochromocytoma surgery, achieving AUC values of 0.766 and 0.767 in the training and validation sets, respectively. Similar to our study, coronary heart disease was an important predictor. Two potential mechanisms may explain its predictive importance; prolonged exposure of the myocardium and coronary arteries to abnormally high catecholamine levels leads to collagen deposition and myocardial fibrosis [13] and Blood Pressure Variability (BPV), which is elevated in PPGL patients, particularly those with excess catecholamine states [32]. Myocardial ischemia and atherosclerosis are common cardiovascular complications in PPGL patients [33], and increased artery stiffness reduces baroreceptor sensitivity, thereby augmenting BPV [34]. Zhang *et al*. [14] developed and validated a nomogram incorporating multiple preoperative CT radiological and clinical parameters (age, tumor shape, BMI, laterality, Mayo Adhesive Probability (MAP) score, and necrosis on CT) based on 112 patients with pheochromocytoma. Their radiology-clinical model (AUC 0.739) outperformed the clinical model (AUC 0.694) in predicting intraoperative hemodynamic instability. However, these two models were limited to pheochromocytomas, did not incorporate radiomic features, and lacked external validation.

Our study has introduced a CT radiomics nomogram, integrating radiomic signatures from contrast-enhanced CT images and clinical factors, like coronary heart disease. These radiomic features have captured intricate tumor heterogeneity and texture not discernible by standard imaging. By incorporating them, our model has achieved higher AUC values (0.91, 0.93, 0.85 for training, validation, and test sets, respectively). This has underscored the enhanced predictive capacity of our approach. The advantages have included non-invasiveness, high accuracy, and ease of use. CT radiomics analysis has offered a comprehensive tumor characterization without patient burden. Furthermore, our nomogram could aid surgeons and anesthesiologists in informed preoperative planning, potentially minimizing intraoperative cardiovascular complications.

Besides showing high predictive accuracy, our CT radiomics nomogram's key aspect is its clinical applicability. Easily incorporated into clinical routines, it could enhance preoperative planning and management for abdominal PPGL patients. Surgeons and anesthesiologists can utilize it to assess intraoperative hypertensive crisis risks using clinical information (*e.g*., coronary heart disease history) and CT data, enabling personalized preoperative preparation, such as optimizing drug regimens and preparing for hemodynamic instability. Accessible via a user-friendly interface or mobile/web app, the nomogram can be used in clinical settings. It can also act as a decision aid for clinicians, informing surgical timing and approach. With high AUC values in validations, our nomogram exhibits promise to improve surgical outcomes and reduce cardiovascular complications in abdominal PPGL patients, although larger studies are needed to confirm these findings and assess long-term impacts.

Our study, however, has some limitations. First, this was a retrospective study conducted at two medical centers. Second, due to the relatively small number of patients with hypertensive crisis, the results may be biased, resulting in a low positive predictive value in the training set model. Future studies with larger sample sizes are necessary to validate our findings and enhance their generalizability. Thirdly, preoperative catecholamines and their metabolites were not included due to their absence in some patients, although our prediction model still exhibited good predictive ability. Fourthly, the manual delineation of VOIs is challenging in the real world, and future studies should focus on developing an automatic segmentation model. Lastly, deep learning features were not used in this study; future work could explore the use of deep learning algorithms to optimize feature selection and improve predictive accuracy.

## CONCLUSION

In conclusion, our developed and validated CT radiomics nomogram has offered advantages over previous methods for predicting intraoperative hypertensive crisis in abdominal pheochromocytoma and paraganglioma patients, including non-invasiveness, simplicity, and high accuracy. However, limitations, such as retrospective design, small sample size, manual tumor delineation, and lack of deep learning features, warrant future studies to refine and optimize its clinical applicability.

## Figures and Tables

**Fig. (1):**
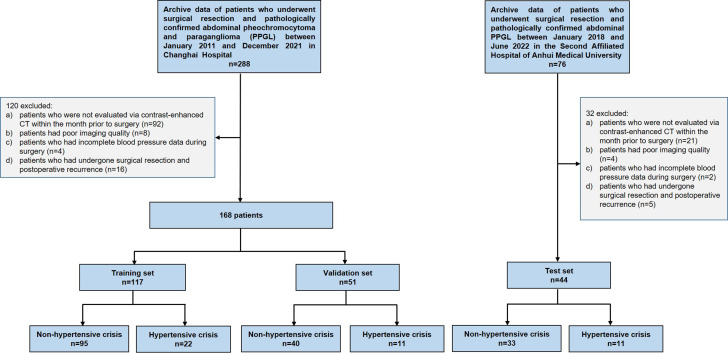
Flowchart depicting the patient selection process.

**Fig. (7):**
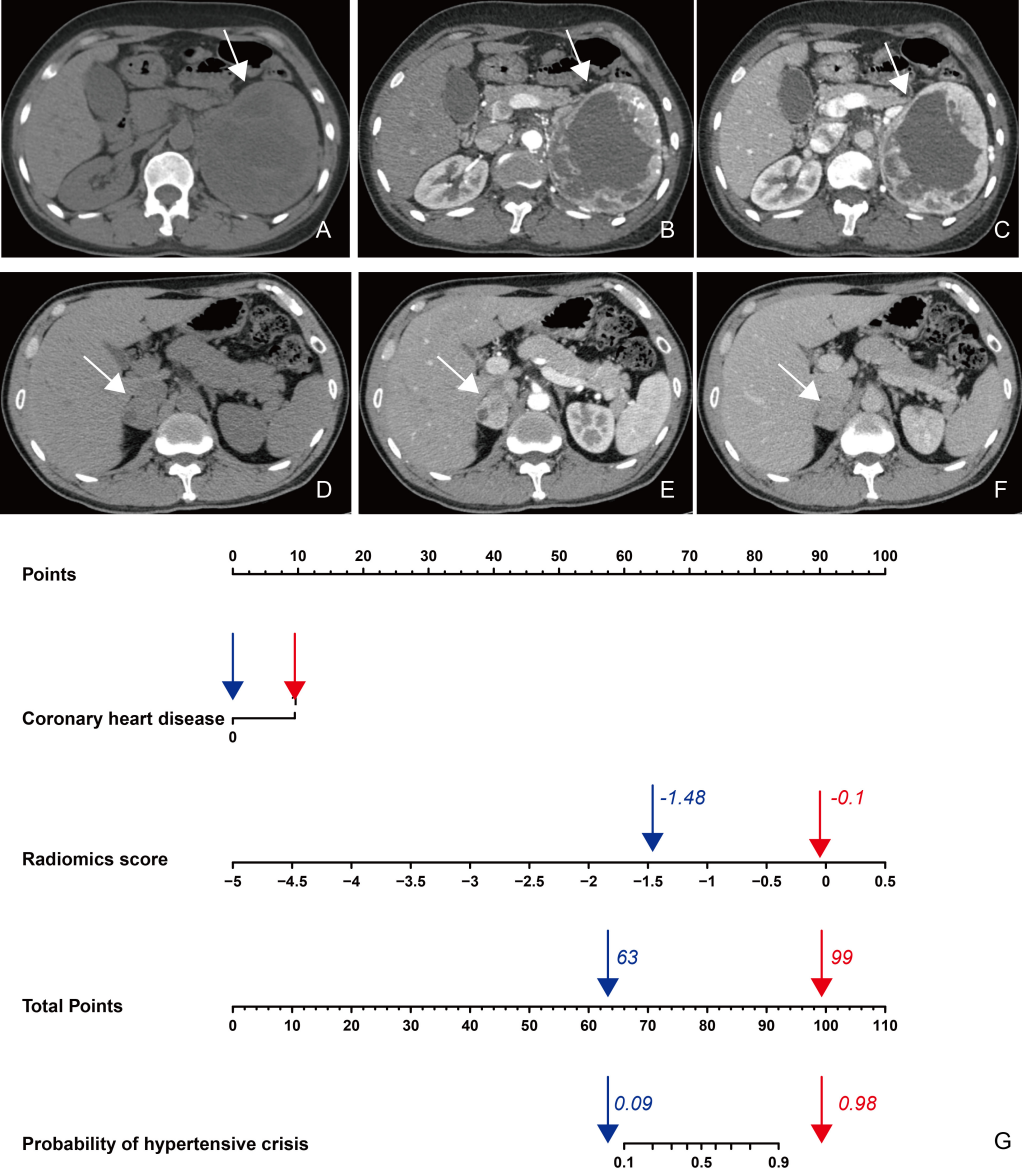
The radiomics nomogram differentiated the patients with pheochromocytoma and paraganglioma and hypertensive crisis from those with non-hypertensive crises. **A-C.** Case 1: A 49-year-old man with pheochromocytoma of the left adrenal gland and non-hypertensive crisis during surgery. **D-F.** Case 2: A 41-year-old woman with pheochromocytoma of the right adrenal gland. She had a history of coronary heart disease and a hypertensive crisis during surgery. **G.** According to the radiomics nomogram, the prediction of hypertensive crisis was 9% (blue arrow) and 98% (red arrow) for cases 1 and 2, respectively.

**Table 1:** Baseline characteristics of patients with abdominal pheochromocytoma and paraganglioma.

Characteristics	Training Set			Validation Set			Test Set		
	Non-hypertensive Crisis Group(n=95)	Hypertensive Crisis Group(n=22)	*p*-value	Non-hypertensive Crisis Group (n=40)	Hypertensive Crisis Group (n=11)	*p*-value	Non-hypertensive Crisis Group (n=33)	Hypertensive Crisis Group (n=11)	*p*-value
**Sex, n (%)**			0.486			0.543			0.484
Male	44 (46.32)	12 (54.55)		21 (52.50)	4 (36.36)		14 (42.42)	6 (54.55)	
Female	51 (53.68)	10 (45.45)		19 (47.40)	7 (63.64)		19 (57.58)	5 (45.45)	
**Age, year (median, range)**	50.00 (20.00, 75.00)	56.50 (28.00,72.00)	0.327	50.00 (24.00, 73.00)	48.00 (36.00,68.00)	0.714	53 (15.00,74.00)	58 (51.00,68.00)	0.052
**BMI, kg/m^2^ (mean ± SD)**	23.03 ± 2.70	23.46 ± 2.81	0.508	24.21 ± 3.93	21.24 ± 2.95	0.024	21.70 ± 2.71	21.70 ± 2.89	1.000
**“Triad” symptoms, n (%)**			0.631			0.828			0.118
No	84 (88.42)	18 (81.82)		36 (90.00)	9 (81.82)		26 (78.79)	6 (54.55)	
Yes	11 (11.58)	4 (18.18)		4 (10.00)	2 (18.18)		7 (21.21)	5 (45.45)	
**Hypertension, n (%)**			0.231			0.477			0.380
No	48 (50.53)	8 (36.36)		17 (42.50)	6 (54.55)		16 (48.48)	3 (27.27)	
Yes	47 (49.47)	14 (63.64)		23 (57.50)	5 (45.45)		17 (51.52)	8 (72.73)	
**Diabetes, n (%)**			0.631			0.828			1.000
No	84 (88.42)	18 (81.82)		36 (90.00)	9 (81.82)		26 (78.79)	8 (72.73)	
Yes	11 (11.58)	4 (18.18)		4 (10.00)	2 (18.18)		7 (21.21)	3 (27.27)	
**Coronary heart disease, n (%)**			0.061			0.216			0.058
No	91 (95.79)	18 (81.82)		40 (100.00)	10 (90.91)		33 (100.00)	9 (81.82)	
Yes	4 (4.21)	4 (18.18)		0 (0.00)	1 (9.09)		0 (0.00)	2 (18.18)	
**Taking hypertensive drugs before surgery, n (%)**			0.364			0.399			0.601
No	31 (32.63)	5 (22.73)		15 (37.50)	2 (4.17)		17 (51.52)	4 (36.36)	
Yes	64 (67.37)	17 (77.27)		25 (62.50)	9 (81.82)		16 (48.48)	7 (63.64)	
**Operation, n (%)**			0.706			0.645			0.652
Laparotomy	56 (58.95)	12 (54.55)		20 (50.00)	7 (63.64)		5 (15.15)	3 (27.27)	
Laparoscopy	39 (41.05)	10 (45.45)		20 (50.00)	4 (36.36)		28 (84.85)	8 (72.73)	
Time of operation, min (median, range)	120.00 (39.00, 390.00)	130.50 (50.00, 370.00)	0.150	135.00 (50.00, 310.00)	115.00 (80.00, 155.00)	0.139	180.00(65.00, 339.00)	145.00(95.00, 331.00)	0.776
**Location, n (%)**			0.894			0.632			0.829
Adrenal	59 (37.74)	14 (4.69)		25 (28.07)	6 (4.17)		27 (81.82)	8 (72.73)	
Non-adrenal	36 (62.26)	8 (95.31)		15 (71.93)	5 (95.83)		6 (18.18)	3 (27.27)	
**Tumor number, n (%)**			0.592			0.545			1.000
1	89 (93.68)	22(100.00)		37 (84.09)	21 (100.00)		32 (96.97)	11 (100.00)	
≥ 2	6 (6.32)	0 (0.00)		3 (6.82)	0 (0.00)		1 (3.03	0 (0.00)	
**Tumor size, mm (median, range)**	48.80 (14.50, 190.90)	54.65 (27.40, 144.8)	0.078	44.25 (19.00, 114.90)	50.60 (27.30, 92.50)	0.330	53.80 (16.70, 93.50)	59.40 (46.40, 172.30)	0.090
**Shape, n (%)**			0.463			0.292			1.000
Rounded	68 (71.58)	14 (63.64)		24 (60.00)	4 (36.36)		24 (72.73)	8 (72.73)	
Lobulated	27 (28.42)	8 (36.36)		16 (40.00)	7 (63.64)		9 (27.27)	3 (27.27)	
**CT attenuation values of tumor, HU (median, range)**									
Arterial phase	108.40 (24.30, 266.90)	93.15 (52.10, 117.6)	0.229	118.80 (42.80, 237.70)	111.00 (75.80, 212.10)	0.536	104.00 (47.00, 203.50)	94.50 (77.40, 163.20)	0.473
Venous phase	117.30 (43.10, 258.80)	109.7 (65.70, 249.3)	0.663	135.40 (70.10, 344.50)	122.20 (72.80, 247.80)	0.622	103.3 (65.00, 182.30)	129.60 (78.40, 150.70)	0.278
** Cystic degeneration ratio, n (%)**			0.282			1.000			0.241
< 50%	64 (67.37)	18 (81.82)		28 (70.00)	8 (72.73)		22 (66.67)	10 (90.91)	
≥ 50%	31 (32.63)	4 (18.18)		12 (30.00)	3 (27.27)		11 (33.33)	1 (9.09)	
** Calcification, n (%)**			0.491			1.000			1.000
No	78 (82.11)	20 (90.91)		34 (85.00)	9 (81.82)		30 (90.91)	10 (90.91)	
Yes	17 (17.89)	2 (9.01)		6 (15.00)	2 (18.18)		3 (9.09)	1 (9.09)	
** Capsular invasion, n (%)**			0.122			1.000			0.012
No	84 (88.42)	22 (100.00)		38 (95.00)	11(100.00)		33 (100.00)	8 (72.73)	
Yes	11 (11.58)	0 (00.00)		2 (5.00)	0 (00.00)		0 (00.00)	3 (27.27)	
** Vascular invasion, n (%)**			1.000			1.000			0.170
No	83 (87.37)	19 (86.36)		33 (82.50)	9 (81.82)		31 (93.94)	8 (72.73)	
Yes	12 (12.63)	3 (13.64)		7 (17.50)	2 (18.18)		2 (6.06)	3 (27.27)	
** Collateral vessel, n (%)**			1.000			1.000			0.262
No	77 (81.05)	18 (81.82)		27 (67.5)	8 (72.73)		24 (72.73)	6 (54.55)	
Yes	18 (18.95)	4 (18.18)		13 (32.5)	3 (27.27)		9 (27.27)	5 (45.45)	
** Feeder artery, n (%)**			0.563			0.785			0.829
No	32 (33.68)	6 (27.27)		15 (37.50)	3 (27.27)		27 (81.82)	8 (72.73)	
Yes	63 (66.32)	16 (72.73)		25 (62.50)	8 (72.73)		6 (18.18)	3 (27.27)	
** Draining vein, n (%)**			0.863			0.833			0.300
No	75 (78.95)	17 (77.27)		33 (82.50)	10 (90.91)		32 (96.97)	9 (81.82)	
Yes	20 (21,05)	5 (22.73)		7 (17.50)	1 (9.09)		1 (3.03)	2 (18.18)	

BMI, body mass index; CT, computer tomography.

**Table 2:** The univariable logistic regression between variables and hypertensive crisis.

Variables	OR (95%CI)	*p-*value
** Sex**		
Male	1.0	
Female	0.72 (0.28, 1.82)	0.487
** Age**	1.02 (0.98, 1.06)	0.285
** BMI**	1.06 (0.89, 1.26)	0.505
**“Triad” symptoms**		
No	1.0	
Yes	1.70 (0.48, 5.94)	0.408
** Hypertension**		
No	1.0	
Yes	1.79 (0.69, 4.65)	0.235
** Diabetes**		
No	1.0	
Yes	1.70 (0.48, 5.94)	0.408
**Coronary heart disease**		
No	1.0	
Yes	5.06 (1.16, 22.10)	0.031
**Taking hypertensive drugs before surgery**		
No	1.0	
Yes	1.65 (0.56, 4.88)	0.368
** Location**		
Adrenal	1.0	
Non-adrenal	0.94 (0.36, 2.45)	0.894
Tumor size	1.01 (0.99, 1.02)	0.265
** Shape**		
Rounded	1.0	
Lobulated	1.44 (0.54, 3.82)	0.465
**CT attenuation values of tumor**		
Arterial phase	0.99 (0.98, 1.00)	0.150
Venous phase	1.00 (0.99, 1.01)	0.751
**Cystic degeneration ratio**		
< 50%	1.0	
≥ 50%	0.46 (0.14, 1.47)	0.190
** Calcification**		
No	1.0	
Yes	0.46 (0.10, 2.15)	0.323
**Capsular invasion**		
No	1.0	
Yes	0.00 (0.00, Inf)	0.989
**Vascular invasion**		
No	1.0	
Yes	1.09 (0.28, 4.25)	0.899
**Collateral vessel**		
No	1.0	
Yes	0.95 (0.29, 3.15)	0.934
**Feeder artery**		
No	1.0	
Yes	1.35 (0.48, 3.79)	0.564
**Draining vein**		
No	1.0	
Yes	1.10 (0.36, 3.36)	0.863
**Radiomics score**	22.20 (5.68, 86.75)	<0.0001

**Note**: OR odds ratio, CI confidence interval; BMI, body mass index; CT, computer tomography.

**Table 3:** Risk factors for hypertensive crisis of abdominal pheochromocytoma and paraganglioma.

Variable	Radiomics Model	
	OR (95% CI)	*p*-value
(Intercept)	9.55 (1.97,46.26)	0.005
Coronary heart disease	4.97 (0.56, 44.21)	0.150
Radiomics score	21.49 (5.37, 85.97)	0.0001

OR, odds ratio; CI, confidence interval

**Table 4:** The performance of the prediction model.

Performance	Radiomics Model		
	Training Set	Validation Set	Test Set
AUC (95% CI)	0.91 (0.85-0.97)	0.93 (0.84-0.99)	0.85 (0.72-0.97)
Sensitivity (%)	86.36	90.91	63.64
Specificity (%)	85.26	90.00	93.94
Accuracy (%)	85.47	90.20	86.36
PPV (%)	57.58	71.43	77.78
NPV (%)	96.43	97.30	88.57

**Note**: AUC, area under the curve; CI, confidence interval; PPV, positive predictive value; NPV, negative predictive value.

## Data Availability

All data generated or analyzed during this study are included in this published article.

## LIST OF ABBREVIATIONS

ADC: Apparent Diffusion Coefficient
AIC: Akaike Information Criterion
AUC: Area Under the Curve
BMI: Body Mass Index
BPV: Blood Pressure Variability
CI: Confidence Interval
CT: Computed Tomography
DCA: Decision Curve Analysis
FOV: Field of View
ICC: Intraclass Correlation Coefficient
LASSO: Least Absolute Shrinkage and Selection Operator
NPV: Negative Predictive Value
OR: Odds Ratio
PPG: Pheochromocytoma and Paraganglioma
PPV: Positive Predictive Value
Rad core: Radiomics Score
ROI: Regions of Interest
VOI: Volume of Interest

## STANDARDS OF REPORTING

STROBE guidelines were followed.
